# Transcriptional and epigenetic regulation of *PPARγ* expression during adipogenesis

**DOI:** 10.1186/2045-3701-4-29

**Published:** 2014-05-29

**Authors:** Ji-Eun Lee, Kai Ge

**Affiliations:** 1 Adipocyte Biology and Gene Regulation Section, Laboratory of Endocrinology and Receptor Biology, National Institute of Diabetes and Digestive and Kidney Diseases, National Institutes of Health, Bethesda, MD 20892, USA

**Keywords:** PPARγ, Adipogenesis, Transcriptional regulation, Enhancer, Epigenetic regulation, Histone acetylation, Histone methylation, Chromatin remodeling

## Abstract

The nuclear receptor PPARγ is a master regulator of adipogenesis. PPARγ is highly expressed in adipose tissues and its expression is markedly induced during adipogenesis. In this review, we describe the current knowledge, as well as future directions, on transcriptional and epigenetic regulation of *PPARγ* expression during adipogenesis. Investigating the molecular mechanisms that control *PPARγ* expression during adipogenesis is critical for understanding the development of white and brown adipose tissues, as well as pathological conditions such as obesity and diabetes. The robust induction of *PPARγ* expression during adipogenesis also serves as an excellent model system for studying transcriptional and epigenetic regulation of cell-type-specific gene expression.

## Introduction

### PPARγ and adipogenesis

**Figure 1:**
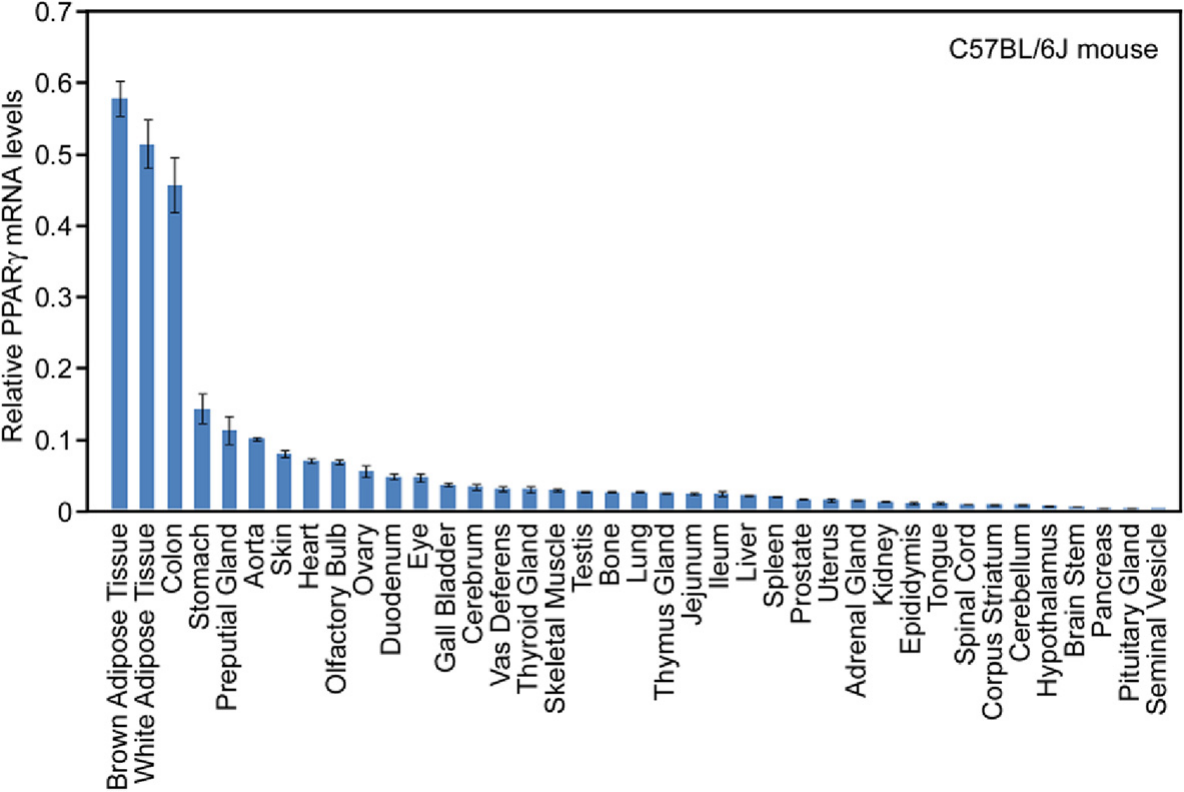
**Tissue distribution of PPARγ.***PPARγ* is highly expressed in brown and white adipose tissues and colon in C57BL/6 J mice. Quantitative reverse-transcriptase PCR (qRT-PCR) of *PPARγ* mRNA levels in various mouse tissues is shown. The original data was obtained from http://www.nursa.org/10.1621/datasets.02001 and modified.

PPARγ (Peroxisome proliferator-activated receptor γ) is a member of the nuclear receptor superfamily of ligand-activated transcription factors (TFs) [1,2]. It is highly expressed in white and brown adipose tissues (Figure 1). PPARγ is considered a master regulator of adipocyte differentiation (adipogenesis) [3]. Ectopic expression of PPARγ in non-adipogenic embryonic fibroblasts stimulates the adipocyte gene transcription program and drives adipogenesis [4]. PPARγ is essential for adipogenesis, as no single factor has been identified that can drive adipogenesis in the absence of PPARγ [5,6]. Consistently, *PPARγ* knockout mice lack terminally differentiated adipose tissues and develop fatty liver and lipodystrophy [7,8].

PPARγ is not only critical for adipogenesis but also important for the maintenance of the fully differentiated state both in culture and in mice [9,10]. Consistently, mutations of the *PPARγ* gene have been implicated in lipodystrophy as well as other metabolic diseases such as hypertension and insulin resistance in humans [11-13]. Antidiabetic insulin-sensitizing drug thiazolidinediones (TZDs) such as Rosiglitasone have been identified as potent and selective ligands of PPARγ [14] but these drugs have undesirable side effects [15].

### *PPARγ* gene

The mouse *PPARγ* gene spans over 120 kb of the genomic sequence on chromosome 6 [16]. It is expressed as two isoforms, PPARγ1 and PPARγ2, as a result of different promoter usage and alternative splicing [16,17]. The *PPARγ1* promoter is located 60 kb upstream of the *PPARγ2* promoter. *PPARγ2* expression is restricted to adipose tissues, while *PPARγ1* is also expressed in various other tissues. Both *PPARγ1* and *PPARγ2* are strongly induced during adipogenesis but are differentially regulated. During adipogenesis of the widely used mouse white preadipocyte cell line 3T3-L1, PPARγ1 is induced earlier than PPARγ2 but the two isoforms are expressed at similar levels in the late phase of differentiation [18]. During adipogenesis of mouse brown preadipocytes, PPARγ1 is induced in the early phase and remains the dominant isoform while PPARγ2 is induced relatively late and remains the minor isoform throughout differentiation [18,19]. The functional differences of the two PPARγ isoforms in adipogenesis and in mature adipose tissues remain elusive. While the regulation of *PPARγ* expression during adipogenesis has been extensively studied, little is known about the regulation of *PPARγ* expression in non-adipose tissues and cells.

### Epigenetic regulation of gene expression

Eukaryotic genomes are packaged into chromatin whose basic unit is the nucleosome [20]. The nucleosome consists of a histone octamer of four core histones (H2A, H2B, H3, H4) wrapped by DNA. The X-ray structure of the nucleosome reveals that histone tails extend outside of the core region [21]. Histone tails are subjected to various covalent modifications (i.e. acetylation, methylation, and phosphorylation), which play important roles in regulating nucleosome structure and recruitment of chromatin-associated proteins [22]. The presence of the nucleosome prevents gene transcription *in vitro *[23]. Nucleosome occupancy correlates inversely with transcription initiation [24]. Therefore, dynamic changes in nucleosome structure are necessary to achieve gene expression. Chromatin remodeling and histone modification are two major epigenetic mechanisms that alter nucleosome structure to regulate gene expression.

#### ***Chromatin remodeling***

Chromatin represses transcription by blocking protein access to the DNA template. Therefore, DNA binding of TFs and transcription cofactors often occurs concurrently with chromatin structure alteration by chromatin remodeling complexes [25]. Two major types of chromatin remodeling complexes have been identified-SWI/SNF and ISWI, both of which contain the ATPase subunit. SWI/SNF complexes disrupt nucleosome core conformation by altering the histone-DNA binding [26]. On the other hand, ISWI complexes promote nucleosome sliding without displacing the histone octamer from DNA [27]. Despite the mechanistic differences, both complexes use the energy from ATP hydrolysis to change nucleosome conformation or location [25].

#### ***Histone modification***

Histones, particularly their N-terminal tails, are covalently modified at many lysine (K) or arginine (R) residues [28]. The combination of covalent modifications affects chromatin structure and gene expression [29]. Acetylation and methylation are two types of extensively studied histone modifications. Histone acetylation is generally correlated with gene activation although it remains to be determined whether a specific histone acetylation is a cause or consequence of gene activation [30]. Acetylation on histones is written by histone acetyltransferases (HATs) and erased by histone deacetylases (HDACs) [31,32]. Recent publications suggest that HATs are highly site-specific in mammalian cells [30,33,34].

Genome-wide profiling by ChIP-Seq reveals that histone methylation correlates with gene activation or repression depending on the methylation sites and states (me1, me2 and me3, i.e. mono-, di- and tri-methylation) [35,36]. Methylation of K4, K36, K79 on histone H3 (H3K4, H3K36, H3K79) correlates with gene activation, whereas di-methylation of K9 or tri-methylation of K27 on H3 (H3K9me2 or H3K27me3) correlate with gene repression. Each modification shows a distinct profile along the genome. For example, tri-methylation on H3K4 (H3K4me3) usually occurs at promoters of actively transcribed genes [37]. Promoter-distal mono- and di-methylation of H3K4 (H3K4me1/2) mark enhancers [38]. Tri-methylation of H3K36 (H3K36me3) associates with elongating RNA polymerase II (Pol II) and is thus enriched on the gene body with peaks at the 3′ end of transcribed regions [35].

Histone methylations are dynamically regulated by remarkably site-specific methyltransferases and demethylases. Histone methyltransferases for H3K4, H3K9, H3K27, H3K36 and H3K79, as well as histone demethylases for H3K4, H3K9, H3K27 and H3K36, have been identified [39,40]. However, their biological functions are largely unclear.

## Transcriptional regulation of *PPARγ* expression during adipogenesis

**Figure 2:**
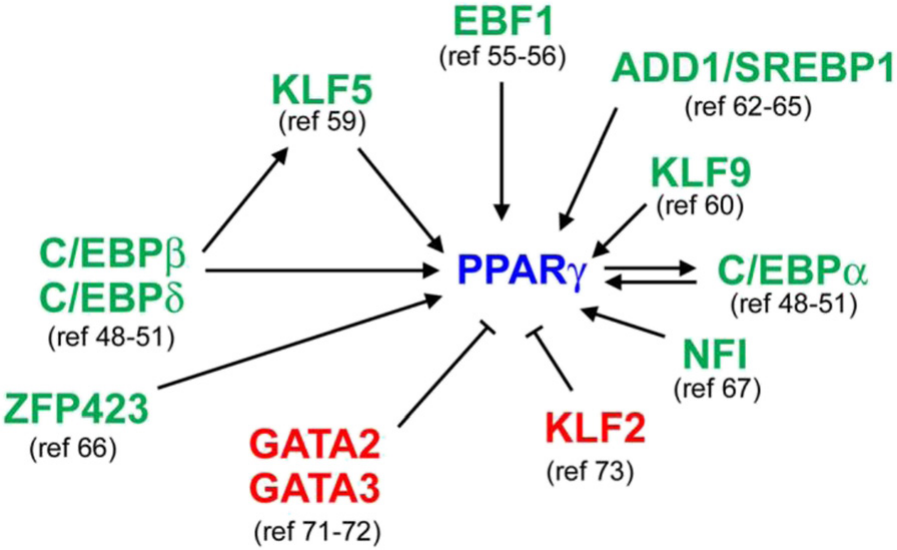
**Transcriptional regulation of *PPARγ *expression during adipogenesis. ***PPARγ* expression is regulated by multiple positive and negative transcription factors (TFs) as well as signaling pathways.

A good number of TFs have been reported to positively or negatively regulate adipogenesis and *PPARγ* expression (Figure 2) [5,6]. However, whether these factors directly regulate *PPARγ* expression is often unclear.

### Positive regulators of *PPARγ* expression

Pro-adipogenic TFs promote adipogenesis in part by directly or indirectly up-regulating *PPARγ* expression or by stimulating PPARγ transcriptional activity. Here, we focus on the factors that have been shown to bind the *PPARγ* gene locus and/or activate the *PPARγ* promoter in reporter assays. It is likely that these factors regulate *PPARγ* expression directly.

#### ***CCAAT/enhancer-binding proteins (C/EBPs)***

C/EBPs, including C/EBPα, β and δ, are basic leucine zipper family TFs that are crucial for adipogenesis [6,41]. C/EBPs form homo- and hetero-dimers to bind to their consensus sequences on target genes [42]. In the early phase of adipogenesis, C/EBPβ and C/EBPδ are induced immediately by adipogenic chemicals isobutylmethylxanthine (IBMX) and dexamethasone (DEX), respectively [43]. Ectopic expression of C/EBPβ alone or together with C/EBPδ induces *PPARγ* expression in non-adipogenic NIH3T3 fibroblasts [44,45]. Conversely, double knockout of *C/EBPβ* and *C/EBPδ* in mice reduces adipose tissue weight [46]. C/EBPα is another adipogenic TF and is both necessary and sufficient for adipogenesis [6,41]. *C/EBPα* expression is induced relatively late around day 2–4 during adipogenesis of L1 cells. C/EBPα knockout mice lack white adipose tissue and show reduced brown adipose tissue [47].

The mouse *PPARγ2* promoter contains two C/EBP recognition elements at -340 bp and -327 bp from the transcription start site [16]. While all three C/EBPs can bind directly to these elements and induce *PPARγ2* expression, C/EBPα binding replaces early C/EBPs at later stages, which is consistent with their expression patterns [48,49]. ChIP-Seq analyses show that C/EBPα, C/EBPβ and PPARγ also bind to enhancer-like regions in the 3′ of the *PPARγ* gene locus [50].

Recent ChIP-Seq analyses reveal that C/EBPβ functions as a pioneer TF in the early phase of adipogenesis [51]. Once C/EBPβ binds to adipogenic enhancer regions (also known as “hotspots”), which can also be found on *PPARγ* and *C/EBPα* gene loci, it facilitates the recruitment of other adipogenic TFs such as glucocorticoid receptor (GR), STAT5A and RXR to form adipogenic enhancers and consequently induces expression of late acting TFs such as PPARγ and C/EBPα [52]. As a pioneer adipogenic TF, C/EBPβ recruits H3K4 mono- and di-methyltransferase MLL4 to establish a subset of active adipogenic enhancers during adipogenesis, including the ones on the *PPARγ* gene locus [50].

#### ***Early B-cell factors (EBFs)***

EBF1 is one of the critical B cell fate determining factors [53]. EBF2 is known to regulate osteoclast differentiation [54]. Both EBF1 and EBF2 are also induced during adipogenesis of 3T3-L1 white preadipocytes but with different expression patterns [55]. Ectopic expression of either factor in NIH3T3 fibroblasts promotes adipogenesis [55,56].

EBF2 is expressed at higher levels in brown compared to white adipose tissues. It has been shown that EBF2 regulates brown adipocyte-specific *Ucp1*and *Prdm16* expression [57]. Although knockout of the *EBF2* gene in mice does not affect *PPARγ* expression, there might be potential redundancy between EBF1 and EBF2. Our unpublished data suggests that EBF2 directly binds to the *PPARγ* gene locus during brown adipogenesis.

The EBF binding motif is highly enriched in active enhancers of adipogenesis and in brown adipose tissue-specific PPARγ binding sites [50,57]. EBF1 binds to *PPARγ1* promoter with the strongest binding at 1 h, suggesting that EBF1 is one of the early regulators of *PPARγ* expression [55]. Future studies are needed to identify the genomic binding profiles of EBF family members during white and brown adipogenesis. The functional redundancy and specificity of EBF family members in regulation of *PPARγ* expression and adipogenesis also need to be clarified.

#### ***Krüppel-like factors (KLFs)***

Several members of KLF family of zinc-finger TFs, including KLF4, KLF5, KLF9 and KLF15, are induced at various stages of 3 T3-L1 adipogenesis. *KLF4* and *KLF5* mRNA levels are induced in the early phase of adipogenesis and peak at around 2 h and 6 h, respectively [58,59]. *KLF9* and *KLF15* mRNA levels are induced at day 2–4 of 3T3-L1 adipogenesis and peak at around day 6–8 [60,61].

Individual knockdown of KLF4, KLF5, KLF9 and KLF15 has been shown to block adipogenesis of 3 T3-L1 preadipocytes, suggesting that these four KLFs play positive roles in adipogenesis [58-61]. Among them, KLF5 and KLF9 have been shown to directly bind to the *PPARγ2* promoter. KLF5 binds from -340 bp to -260 bp of the *PPARγ2* promoter and cooperates with C/EBPs to induce *PPARγ2* expression [59]. KLF9 binds from -413 bp to -247 bp of the *PPARγ2* promoter and moderately activates the *PPARγ2* promoter in a luciferase reporter assay [60]. Thus, KLF5 and KLF9 show distinct expression patterns during adipogenesis but appear to share the same region on *PPARγ2* promoter. To understand the mechanisms by which KLFs regulate adipogenesis and *PPARγ* expression, the genomic binding profiles of KLFs during adipogenesis need to be determined. The functional redundancy and specificity of KLFs in regulation of adipogenesis and *PPARγ* expression also need to be clarified.

Unlike the KLFs mentioned above, KLF2 has been reported to inhibit *PPARγ* expression and will be discussed in a later section [62].

#### ***Sterol regulatory element-binding protein-1 (SREBP1)***

SREBP1 (also known as ADD-1; adipocyte determination and differentiation factor 1) is a basic helix-loop-helix leucine zipper TF involved in cholesterol homeostasis [63]. It is expressed in various tissue types with the highest level in brown adipose tissue [64]. *SREBP1* is induced during differentiation of 3T3-F442 and 3T3-L1 preadipocytes. A dominant-negative form of SREBP1 with a mutation in the DNA-binding domain markedly inhibits adipogenesis of 3T3-L1 cells [65]. Ectopic expression of SREBP1 induces endogenous *PPARγ* expression in 3T3-L1 and HepG2 cells. SREBP1 has been shown to bind to a putative E-box motif at -1535 bp of the *PPARγ1* promoter, and mutation of this motif inhibits SREBP1 binding [66]. To understand how SREBP1 regulates *PPARγ* expression and adipogenesis, it will be critical to determine the genomic binding profile of SREBP1 during adipogenesis.

#### ***Zinc finger protein 423 (ZFP423)***

ZFP423 is a zinc finger TF and was recently identified as a preadipocyte determination factor [67]. It is enriched in preadipocytes compared to non-adipogenic fibroblasts and its expression levels positively correlate with the adipogenic potential of fibroblasts. Ectopic expression of ZFP423 in non-adipogenic NIH3T3 fibroblasts induces *PPARγ2* but not *PPARγ1* expression before differentiation and promotes adipogenesis after induction of differentiation. Conversely, knockdown of ZFP423 in 3T3-L1 preadipocytes blocks adipogenesis and decreases both *PPARγ1* and *PPARγ2* levels before and after differentiation [67]. However, the molecular mechanism by which ZFP423 regulates *PPARγ* expression remains incompletely understood.

#### ***Nuclear factor I (NFI)***

The NFI family TFs were identified as potential novel regulators of adipogenesis from computational motif analysis of adipocyte-specific open chromatin regions in 3T3-L1 cells [68]. The NFI binding motif also shows up in other studies involving epigenomic profiling of adipogenesis [50,57]. During adipogenesis, NFIA and NFIB expression levels are significantly induced while NFIC and NFIX levels remain steady. Knockdown of either NFIA or NFIB reduces the differentiation ability of 3T3-L1. ChIP analysis reveals binding of NFIA to known distal regulatory elements of *PPARγ* and *C/EBPα*, as well as *Fabp4* genes [68].

### Positive cross-regulation between PPARγ and C/EBPα

**Figure 3:**
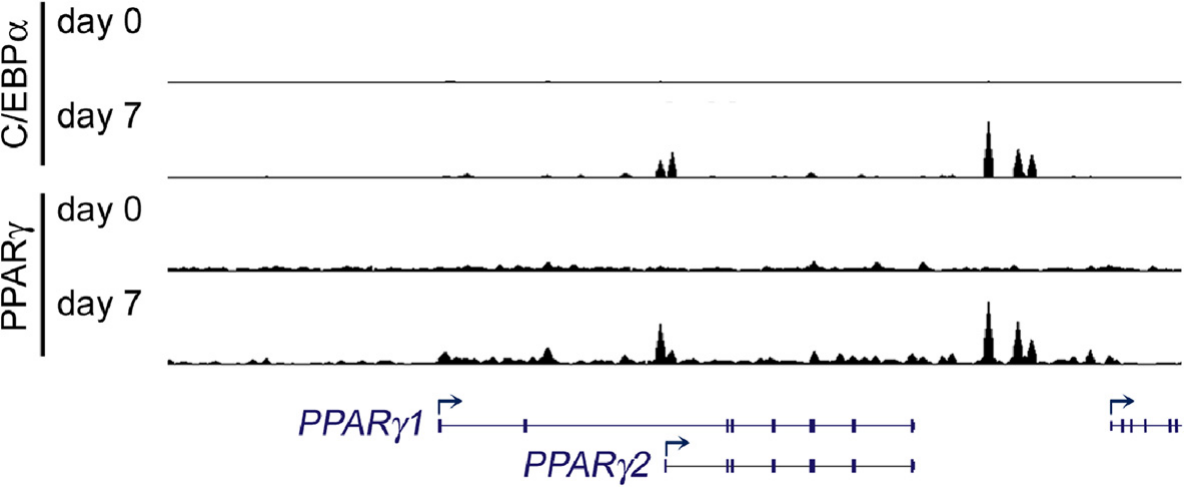
**Co-localization of PPARγ and C/EBPα on *PPARγ *gene locus during adipogenesis.** Snapshot of PPARγ and C/EBPα binding profiles on the *PPARγ* gene locus during brown adipogenesis. ChIP-Seq of PPARγ and C/EBPα were performed before (day 0) and after (day 7) differentiation of immortalized brown preadipocytes (unpublished). Enrichment of peaks was visualized in the UCSC genome browser.

PPARγ and C/EBPα positively regulate each other’s expression and cooperate to promote adipogenesis [3,69]. PPARγ is essential for C/EBPα-stimulated adipogenesis in fibroblasts [3]. Conversely, *C/EBPα* knockout fibroblasts show severe defects in PPARγ-stimulated adipogenesis in the absence of synthetic PPARγ ligands and partial defects in the presence of these ligands, suggesting that C/EBPα is also required for PPARγ-stimulated adipogenesis [19]. PPARγ directly activates endogenous *C/EBPα* gene transcription. Once induced, C/EBPα binds to the *PPARγ* gene locus and further induces and maintains *PPARγ* expression in mature adipocytes through a positive feedback loop [3]. Genome-wide profiling studies show that during adipogenesis, most induced genes are bound by both PPARγ and C/EBPα, suggesting a synergistic upregulation of adipogenic gene expression by these two master regulators [70,71]. Interestingly, PPARγ binds its own gene locus, where C/EBPα also binds (Figure 3). These regions include the *PPARγ2* promoter and downstream intergenic enhancer regions [50].

### Negative regulators of *PPARγ* expression

#### ***GATAs***

Of the six GATA family zinc finger domain TFs, *GATA-2* and *GATA-3* are highly expressed in the preadipocyte fraction of white adipose tissues in mice. Their expression is down-regulated during differentiation of 3T3-F442A preadipocytes. Constitutive expression of GATA-2 or GATA-3 inhibits 3T3-F442A differentiation and *PPARγ* expression. Consistently, GATA-3 knockout ES cells show enhanced ability of differentiation towards adipocytes. In a luciferase reporter assay, GATA-2 and GATA-3 inhibit the activity of 0.6-kb *PPARγ2* proximal promoter [72]. GATA-2 and GATA-3 are also found to inhibit the transcriptional activities of C/EBPα and C/EBPβ through physical interactions, thus offering an additional mechanism by which GATA-2/3 inhibit adipogenesis and *PPARγ* expression [73]. It remains to be determined whether GATA-2/3 directly repress *PPARγ* expression in preadipocytes.

#### ***KLF2***

Overexpression of KLF2 strongly inhibits differentiation of 3T3-L1 preadipocytes and the expression of *PPARγ* but not the early adipogenic TFs *C/EBPβ* and *C/EBPδ*. KLF2 can directly bind to the CACCC element within the *PPARγ2* proximal promoter region and repress *PPARγ2* promoter activity in a reporter assay. However, mutation of its binding site alone is insufficient to block KLF2-mediated repression of the *PPARγ2* promoter, suggesting that other mechanisms are also involved [62]. It remains to be determined whether endogenous KLF2 directly represses *PPARγ* expression in preadipocytes.

## Epigenetic regulation of *PPARγ* expression during adipogenesis

**Figure 4:**
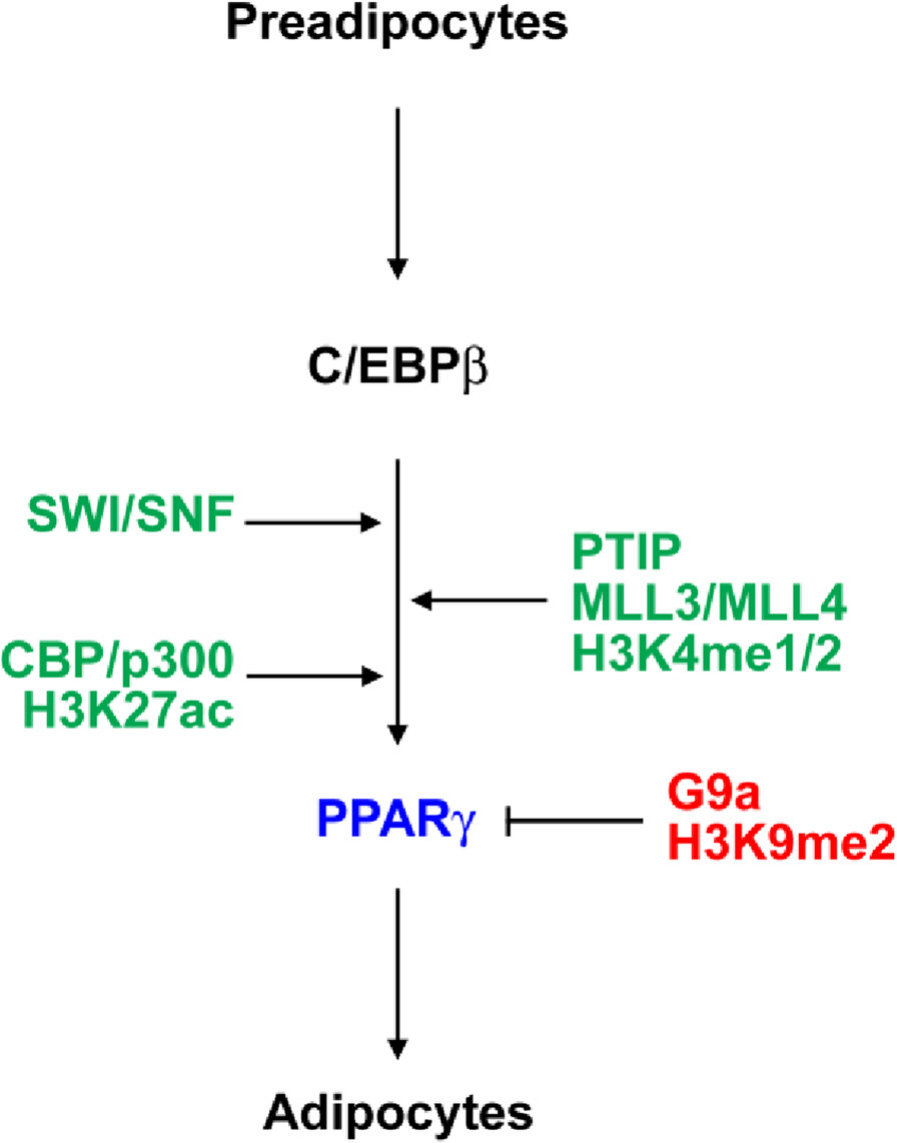
**Positive and negative epigenetic regulators of *PPARγ *** **expression during adipogenesis.**

*PPARγ* expression during adipogenesis is regulated by chromatin remodeling and histone modifications such as acetylation and methylation (Figure 4).

### Chromatin remodeling

The *PPARγ* gene locus undergoes dynamic changes within hours of adipogenesis induction. DNase I hypersensitivity assays reveal that chromatin remodeling and opening of the *PPARγ* gene locus occur within 3–4 hours of induction in 3T3-L1 cells [51]. The major opening regions are the *PPARγ2* promoter and 3′ distal regions that are occupied by C/EBPβ, C/EBPα, and PPARγ itself in later stages of differentiation [50].

Chromatin remodeling and opening at the *PPARγ2* promoter are adipose-specific and dependent on cAMP and protein kinase A (PKA) pathways [74]. IBMX alone can induce chromatin opening of the *PPARγ2* promoter. Conversely, shRNA-mediated knockdown of PKA subunits inhibits chromatin accessibility of the *PPARγ2* promoter region.

The SWI/SNF chromatin remodeling complex has been shown to regulate *PPARγ2* expression during adipogenesis [75]. The dominant-negative form of Brg1, an ATPase subunit of the SWI/SNF complex, inhibits PPARγ-, C/EBPα-, or C/EBPβ-induced adipogenesis in fibroblasts. In the early stage of 3T3-L1 differentiation, C/EBP factors bind to the *PPARγ2* promoter, followed by Pol II and general TFs assembly prior to and independently of SWI/SNF. SWI/SNF and TFIIH then assemble on the *PPARγ2* promoter to facilitate preinitiation complex formation. It remains unclear how the SWI/SNF complex is recruited to the *PPARγ* gene locus.

### Histone acetylation

Histone acetylations generally correlate with gene activation and are catalyzed by site-specific histone acetyltransferases (HATs). In mammalian cells, the homologous and functionally redundant HATs GCN5 and PCAF specifically acetylate H3K9, while another pair of homologous and functionally redundant HATs CBP and p300 specifically acetylates H3K18 and H3K27 [30].

**Figure 5:**
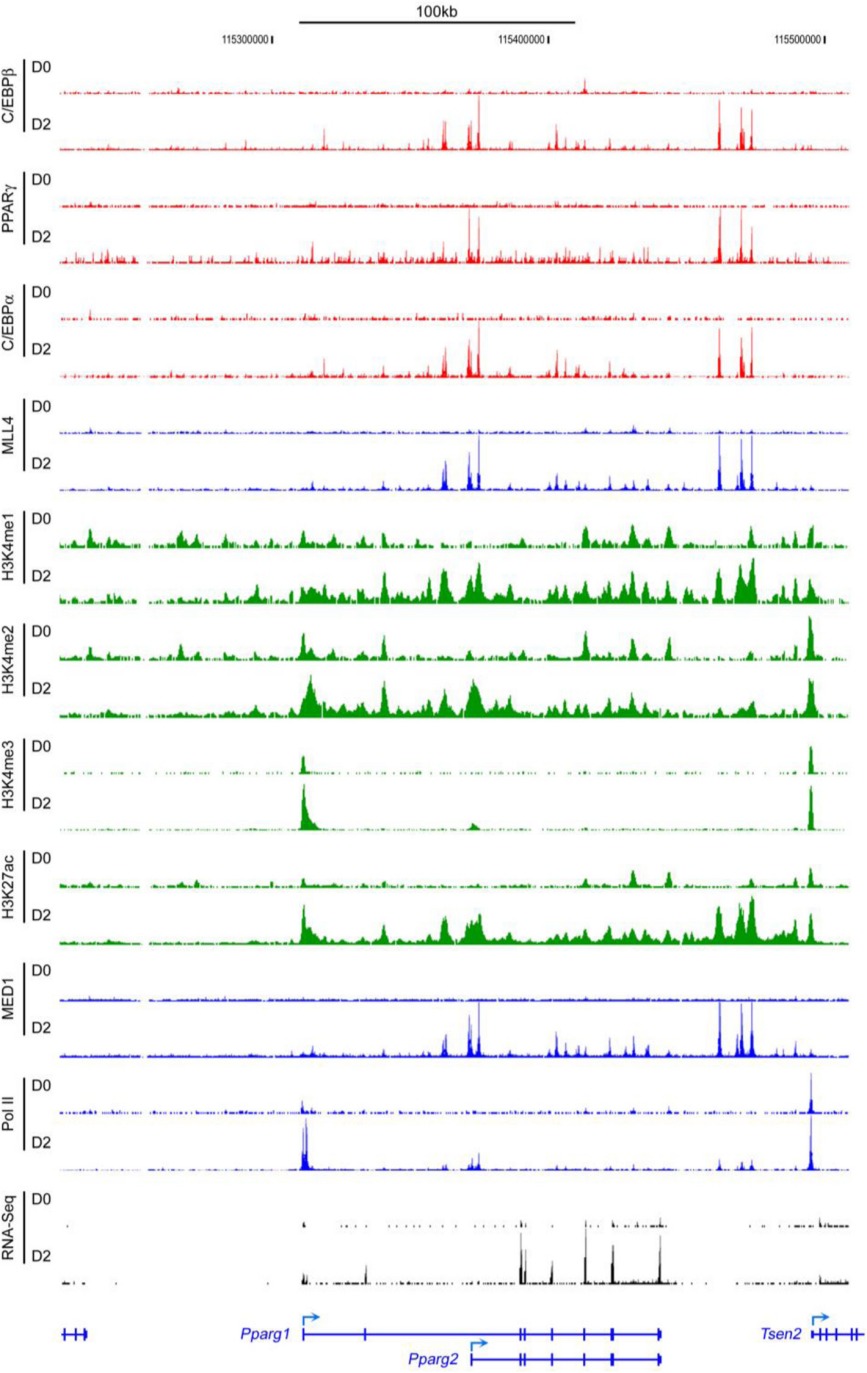
**ChIP-Seq and RNA-Seq profiles on *PPARγ *gene locus during adipogenesis.** ChIP-Seq and RNA-Seq were performed during brown preadipocyte differentiation. The original sequencing data was obtained from NCBI GEO database (http://www.ncbi.nlm.nih.gov/geo/) under accession number GSE50466 [50].

Genome-wide profiling of H3K9ac and H3K27ac reveals that both marks are highly induced on the *PPARγ* gene locus during 3T3-L1 differentiation and correlate with *PPARγ* gene expression [76,77]. While the roles of GCN5/PCAF and GCN5/PCAF-mediated H3K9ac in regulating *PPARγ* expression and adipogenesis remain to be determined, CBP/p300 are known to be essential for adipogenesis and *PPARγ* expression [78]. CBP/p300-mediated H3K27ac is a marker for active enhancers and therefore highly associates with cell type-specific gene expression [79]. ChIP-Seq of H3K27ac has revealed adipose-specific active enhancers located in the intergenic regions downstream of the *PPARγ* gene (Figure 5) [50]. The functional roles of these enhancers in regulating *PPARγ* expression during adipogenesis remain to be examined.

HBO1, also known as MYST2 or KAT7, is a member of the MYST family of HATs. It is specifically required for H3K14ac in mammalian cells [34]. Another member of the MYST family of HATs, MOF (also known as MYST1 or KAT8), is specifically required for H4K16ac in mammalian cells [80]. Both H3K14ac and H4K16ac positively correlate with gene expression. However, the roles of HBO1-mediated H3K14ac and MOF-mediated H4K16ac in regulating *PPARγ* expression and adipogenesis remain to be determined.

### Histone methylation

Several histone methyltransferases and demethylases have been shown to regulate adipogenesis [19,39,50,81-83]. Among them, H3K4 mono- and di-methyltransferases MLL3 (KMT2C) and MLL4 (KMT2D) directly promote *PPARγ* expression during adipogenesis [50]. In contrast, H3K9 mono- and di-methyltransferase G9a (EHMT2) directly represses *PPARγ* expression in preadipocytes and during adipogenesis [81]. While H3K27 methyltransferase Ezh2 directly represses *Wnt* genes to facilitate adipogenesis, Ezh2 and Ezh2-mediated H3K27me3 are absent from the *PPARγ* gene locus during adipogenesis and thus promote *PPARγ* expression indirectly [83].

#### ***H3K4 methyltransferases MLL3/MLL4 directly promote PPARγ expression***

Homologous MLL3 and MLL4 belong to the mammalian SET1-like H3K4 methyltransferase family [84-86]. Each member of this family of six methyltransferases forms a large protein complex that contains WDR5, RbBP5, ASH2L, and DPY30 (WRAD) subunits [84-87]. In addition to the common WRAD subunits, MLL3/MLL4 complexes also contain unique subunits, including H3K27 demethylase UTX, nuclear receptor coactivator NCOA6, BRCT domain-containing protein PTIP, and a novel protein PA1 (also known as PAGR1) [84,88-90].

Enhancers control cell-type-specific gene expression and are marked with H3K4me1/2 [38]. We recently showed that MLL3 and MLL4 are major H3K4 mono- and di-methyltransferases in human and mouse cells. ChIP-Seq analyses reveal that MLL4 shows cell-type- and differentiation-stage-specific genomic binding and is mainly enriched on active enhancers during cell differentiation. MLL3 and MLL4 are partially redundant and are major H3K4 mono- and di-methyltransferases on enhancers. Using adipogenesis and myogenesis as model systems, we showed that MLL3 and MLL4 are required for enhancer activation, cell-type-specific gene induction and cell differentiation [50].

MLL3 and MLL4 have partially overlapping functions and are essential for *PPARγ* expression and adipogenesis [50]. During adipogenesis, MLL4 is mainly enriched on adipogenic enhancers, which are active enhancers bound by PPARγ, C/EBPα and C/EBPβ. MLL4 physically interacts with C/EBPβ and PPARγ in cells. In the early phase of adipogenesis, the pioneer adipogenic TF C/EBPβ recruits MLL4 to perform H3K4me1/2 and establish adipogenic enhancers on gene loci encoding the master adipogenic TFs PPARγ (Figure 5) and C/EBPα. After PPARγ and C/EBPα are induced, they recruit MLL4 to perform H3K4me1/2 and establish enhancers critical for adipocyte gene expression. Deletion of MLL3 and MLL4 in preadipocytes prevents the activation of adipogenic enhancers on *PPARγ* and *C/EBPα* genes, as well as their induction, which lead to severe defects in adipogenesis [50]. MLL4 appears to be the major regulator of adipogenesis in mice with MLL3 playing a minor role [50,91]. Knockout of MLL4 by muscle- and brown adipose tissue (BAT)-selective Myf5-Cre in mice inhibits normal development of Myf5+ muscles and BAT.

MLL3/MLL4-associated NCOA6 and PTIP have also been shown to be important for adipogenesis, although the underlying molecular mechanisms are unclear [19,92]. PTIP is a nuclear protein with functions in transcription and DNA damage response. PTIP directly controls the induction of *PPARγ* and *C/EBPα* during adipogenesis and is required for the enrichment of MLL3/MLL4 complexes on the *PPARγ* promoter region [19]. Since PTIP itself does not possess a DNA binding domain, PTIP may function through MLL3/MLL4 to help establish adipogenic enhancers. This hypothesis can be tested by performing ChIP-Seq of PTIP in MLL3/MLL4 knockout cells and vice versa.

#### ***H3K9 methyltransferase G9a directly represses PPARγ expression***

H3K9me2 and H3K27me3 are two major repressive epigenetic marks. G9a is the major methyltransferase responsible for H3K9me2 while Ezh2 is the major methyltransferase responsible for H3K27me3 in cells [93-95]. ChIP-Seq reveals that the genomic locations of H3K9me2 and H3K27me3 are largely non-overlapping in preadipocytes [81]. On gene loci encoding the major negative and positive regulators of adipogenesis, *Wnt* gene loci are marked with high levels of H3K27me3 but little H3K9me2. In contrast, the entire *PPARγ* gene locus is covered with high levels of H3K9me2 but little H3K27me3. H3K9me2 levels are also low on gene loci encoding other positive regulators of adipogenesis, including C/EBPs, KLF4, Krox20 and CREB. During adipogenesis, H3K9me2 levels and G9a protein levels decrease significantly. Deletion of G9a in preadipocytes or inhibition of G9a methyltransferase activity increases *PPARγ* expression and adipogenesis by promoting C/EBPβ binding to and chromatin opening of the *PPARγ* gene locus [81].

The inverse correlation between the genomic locations of H3K9me2 and H3K27me3 in preadipocytes suggests that G9a-mediated H3K9me2 is a major repressive epigenetic mechanism that regulates *PPARγ* expression in the early phase of adipogenesis. The marked decrease of H3K9me2 on the entire *PPARγ* gene locus during adipogenesis suggests that H3K9me2 demethylases may antagonize G9a function. Among the known H3K9 demethylases [39], it is currently unknown which H3K9 demethylases remove H3K9me2 on the *PPARγ* gene locus during adipogenesis.

## Future directions

In addition to identifying novel regulators of *PPARγ* expression during adipogenesis, future studies should at least address the following issues: i) distinguishing direct vs. indirect regulators of the *PPARγ* gene, ii) characterizing of putative *PPARγ* enhancers, iii) understanding the mechanisms by which epigenetic regulators are recruited to the *PPARγ* gene locus, and iv) determining of chromatin interaction of the *PPARγ* gene locus.

### Distinguishing direct vs. indirect regulators of *PPARγ* gene

A good number of TFs and epigenetic factors have been shown to modulate adipogenesis and the associated *PPARγ* expression [96]. However, it is largely unclear whether these factors directly or indirectly regulate the *PPARγ* gene. Genome-wide profiling by ChIP-Seq has made it possible to map genomic binding sites of these factors and to distinguish direct vs. indirect regulators of *PPARγ* expression during adipogenesis in an unbiased way. Genome-wide binding profiles of PPARγ, C/EBPα, C/EBPβ during adipogenesis have been generated [50,68,70,71,76]. The genomic binding sites of other adipogenic TFs including EBFs, KLFs, SREBP1, ZFP423 and NFI, as well as the genomic binding sites of negative regulators of *PPARγ* expression and adipogenesis including GATA2/3 and KLF2, need to be determined using ChIP-Seq.

### Characterization of putative *PPARγ* enhancers

Enhancers regulate cell-type-specific gene expression and promote gene transcription by delivering necessary factors to the promoters. The interaction between enhancers and promoters is critical for cell-type-specific gene transcriptional programs [38]. Therefore, identifying cell-type-specific enhancers is important for understanding the mechanisms that control the expression of developmental genes. ChIP-Seq profiling of adipogenic TFs PPARγ and C/EBPα/β, enhancer marks H3K4me1/2 and the H3K4me1/2 methyltransferase MLL4, as well as active enhancer mark H3K27ac, has enabled the identification of putative adipocyte-specific enhancers in the intergenic region downstream of *PPARγ* gene (Figures 3 and 5).

The next step is to characterize and validate these putative *PPARγ* enhancers. The traditional luciferase reporter assay is easy to perform but has several limitations [97]. First, the enhancer DNA sequence cloned into the luciferase reporter plasmid lacks the native chromatin structure and therefore may not be representative of the physiological enhancer. Second, most genes are regulated by multiple enhancers, but only one enhancer can be tested in a reporter assay. The recently developed genome editing technique CRISPR could be a better approach [98]. This method can be used to disrupt *PPARγ* enhancers in preadipocytes to validate their functional importance in regulating *PPARγ* expression during adipogenesis.

### How are epigenetic factors recruited to the *PPARγ* gene locus?

Sequence-specific TFs likely play a major role in the recruitment of epigenetic factors to target gene loci because most epigenetic regulators lack DNA-binding domains. For example, the MLL3/MLL4 complexes physically interact with PPARγ and C/EBPβ [50,91]. Ectopic expression of C/EBPβ alone in undifferentiated preadipocytes is sufficient to recruit MLL4, MLL4-mediated H3K4me1, and active enhancer mark H3K27ac to a subset of enhancers on the *PPARγ* gene locus. This suggests that C/EBPβ, likely in cooperation with PPARγ and C/EBPα, recruits the MLL4 complex to establish active *PPARγ* enhancers to promote *PPARγ* expression during adipogenesis. The factors that recruit H3K9 methyltransferase G9a to directly repress *PPARγ* gene remain to be identified.

Recent studies suggest that long intergenic noncoding RNAs (lncRNAs) can mediate the interactions between epigenetic regulator with genome or with other epigenetic regulators [99,100]. Expression of several lncRNAs is strongly induced during adipogenesis [101]. Whether any of these lncRNAs directly regulates *PPARγ* expression remains to be determined.

### Chromatin interaction of *PPARγ* gene locus

Physical interaction between distal enhancers and the gene promoter is critical for active gene expression, as shown on the β-globin locus [102]. Development of the chromosome conformation capture (3C) assay has enabled the identification of long range chromatin interactions [103]. The limitation of 3C is that we can only see a very narrow region of interest [104]. Updated versions of this technique including 4C and 5C assays involve high-throughput sequencing and therefore provide an unbiased picture of chromatin interaction in given cell types. So far, no study has reported the chromatin interaction of *PPARγ* gene locus and its role in regulation of *PPARγ* expression.

In summary, *PPARγ* expression during adipogenesis provides an excellent model system for understanding the transcriptional and epigenetic regulation of cell-type-specific gene transcription programs and cell differentiation. One of our big challenges is to validate the function of regulatory elements outside of the coding region and identify associated factors and 3D genome structure around the *PPARγ* locus. Such information would be of great help to understanding adipose-related human metabolic diseases, particularly obesity and type II diabetes.

## Abbreviations

cAMP: Cyclic 3′,5′-adenosine monophosphate; CBP: CREB-binding protein; C/EBP: CCAAT/enhancer-binding protein; ChIP-Seq: Chromatin immunoprecipitation sequencing; DEX: Dexamethasone; EBF: Early B-cell factor; EHMT2: Euchromatic histone-lysine N-methyltransferase 2; Ezh2: Enhancer of zeste homolog 2; GATA: GATA-binding protein; GCN5: General control of amino acid synthesis protein 5-like 2; KLFs: Krüppel-like factors; HAT: Histone acetyltransferase; HDAC: Histone deacetylase; IBMX: Isobutylmethylxanthine; KMT: Lysine-specific methyltransferase; MLL3/4: Myeloid/lymphoid or mixed-lineage leukemia 3/4; PA1: PTIP-associated 1 protein; PCAF: p300/CBP-associated factor; PKA: Protein kinase A; PPARγ: Peroxisome proliferator-activated receptor γ; PTIP: PAX transactivation domain-interacting protein; ISWI: Immitation of switch; SREBP1: Sterol regulatory element-binding protein-1; NCOA6: Nuclear receptor coactivator 6; NFI: Nuclear factor I; SWI/SNF: Switch/Sucrose nonfermenting; TF: Transcription factor; WDR5: WD repeat-containing protein 5; ZFP423: Zinc finger protein 423.

## Competing interests

The authors declare that they have no competing interests.

## Authors’ contributions

J-EL carried out the molecular genetic studies, drafted and revised the manuscript. KG drafted and revised the manuscript. Both authors read and approved the final manuscript. This research was supported by the Intramural Research Program of the NIH, The National Institute of Diabetes and Digestive and Kidney Diseases (NIDDK).
